# The impact of wood dust on pulmonary function and blood immunoglobulin E, erythrocyte sedimentation rate, and C‐ reactive protein: A cross‐sectional study among sawmill workers in Tangail, Bangladesh

**DOI:** 10.1002/hsr2.646

**Published:** 2022-05-22

**Authors:** Md. Roman Mogal, Md. Didarul Islam, Md. Ikbal Hasan, Asadullah Junayed, Sagarika Adhikary Sompa, Md. Rashel Mahmod, Aklima Akter, Md. Zainul Abedin, Md. Asaduzzaman Sikder

**Affiliations:** ^1^ Department of Biochemistry and Molecular Biology Mawlana Bhashani Science and Technology University Tangail Bangladesh; ^2^ Department of Food Technology and Nutritional Science Mawlana Bhashani Science and Technology University Bangladesh

**Keywords:** IgE, respiratory health, sawmill workers, spirometry, wood dust

## Abstract

**Background and Aims:**

Occupational exposure to wood dust leads to lung function abnormalities that are prominent causes of morbidity and disability of sawmill workers. The adverse respiratory effects of wood dust in sawmills have not been studied thoroughly in Bangladesh. This study aimed to investigate the effect of wood dust on the respiratory health of sawmill workers compared to controls as well as to determine the association of wood dust‐exposing effects with inflammatory blood biomarkers, such as immunoglobulin E (IgE), erythrocyte sedimentation rate (ESR), and C‐reactive protein (CRP).

**Methods:**

This cross‐sectional study included 100 sawmill workers from 25 distinct sawmills in various areas of Tangail, Bangladesh as well as 100 healthy volunteers who were adopted as a control group. Questionaries' survey and pulmonary function tests were performed face to face. Furthermore, after performing lung function tests, blood was drawn for further IgE, ESR, and CRP analyses.

**Results:**

Respiratory symptoms including breathlessness (32%), coughing (39%), sneezing (43%), chest tightness (30%), and itching (40%) were significantly higher in sawmill workers compared with control. Besides, sawmill workers' exposure to wood dust revealed a significantly lower level of spirometry parameters (forced vital capacity ​​​​​[FVC], FVC (%), forced expiratory volume in 1 s [FEV1], FEV1 (%), peak expiratory flow [PEF], PEF (%), FEV1/FVC (%), FEF25, FEF75, and FEF2575) compared with control and these spirometry parameters decreased with the increasing length of service. Moreover, a significantly higher level of IgE was observed in sawmill workers (290.90 ± 39.49) than in the control (120.95 ± 23.00). The high level of IgE suggests that the lower pulmonary function may be linked to allergic responses to wood dust among sawmill workers.

**Conclusion:**

This study suggested that exposure to wood dust can cause impairment of respiratory function along with high IgE levels.

## 1 INTRODUCTION

Work‐related respiratory disorders afflict workers in all industries, accounting for roughly 60% of all disease and injury mortality and 70% of all occupational disease mortality.^1^ In 2016, based on the Global Burden of Diseases data set, 519,100 deaths and 13.6 million disability incidents occurred from chronic respiratory diseases due to occupational airborne exposure.^2^ Moreover, the European Respiratory Society estimated that more than 50 million people are living with occupational lung diseases.^3^ According to Health and Safety Executive, in Great Britain, 12,000 lung disease deaths and 17,000 new cases are reported each year linked to past exposure in working place.^4^ Workers, insurers, and the general public all bear a substantial financial burden as a result of occupational lung diseases. It was enumerated that the annual cost of occupational exposure‐related disorders, including respiratory diseases, is estimated to be $26 billion.^5^ Moreover, 28 countries in the European Union annually face €42 billion in production loss due to lung diseases.^6^

Exposure to wood dust results in a higher risk of respiratory diseases and increased morbidity.^7^ Two types of woods, hardwood (Angiosperms) and softwood (Gymnosperms), are used for making clipboards, furniture, and woodworking.^8^ Wood dust consisting of cellulose, hemicelluloses, lignin, and small amounts of extraneous materials^9^ is produced as a by‐product when the wood is cut and shaped.^10^ The nature of the job, not adopting personal protective equipment, poor ventilation system, using antediluvian materials, and other factors have been responsible for prolonging dust exposure at sawmills.^11^ Inhalation of wood dust can cause symptoms, including cough, chest tightness, wheezing, dyspnea, sneezing, and headache.^12^, ^13^ It has been documented that exposure to wood dust is associated with several health effects such as rhinitis, chronic bronchitis, asthma, pneumonitis, nasopharyngeal, and other upper and lower respiratory symptoms and the intensity of these conditions augment with increasing exposure time.^13^, ^14^, ^15^ Moreover, wood dust is considered carcinogenic based on human carcinogenicity studies.^16^, ^17^ Furthermore, several studies have concluded that the lung function of sawmill workers associated with wood dust is lower than their counterparts^7^, ^18^, ^19^ which, unfortunately, can drop despite being young.^20^ The abatement of lung function can worsen due to wood dust containing biological organisms, such as endotoxin, Gram‐negative bacteria, allergenic fungi, and mold.^21^, ^22^ Also, biological organisms may lead to hypersensitivity reactions that subsequently lead to asthma.^8^

Having a lot of demand for wood to make furniture and house, many sawmills have been established in Bangladesh. It is enumerated by the Bangladesh Bureau of Statistics that there are 16,138 sawmills in Bangladesh and more than 87,715 people are involved in this sector.^23^ Most of the sawmills in Bangladesh use obsolete machines that produce huge wood dust and the workers do not follow the safety precaution during working. As a result, produced wood dust can probably impair the lung function of the sawmill workers. Henceforth, this study aimed to determine the effects of wood dust on Bangladeshi sawmill workers' lung function and inflammatory blood markers, such as immunoglobulin E (IgE), erythrocyte sedimentation rate (ESR), and C‐reactive protein (CRP).

## 2 MATERIALS AND METHODS

### 2.1 Research design and study population

This cross‐sectional study was conducted on sawmill workers, where workers were occupationally exposed to wood dust particles during cutting wood by machines at their workplace, in different zones of Tangail, Bangladesh from October 2019 to March 2020. For the study purpose, 100 sawmill workers were chosen randomly from 25 distinct sawmills. The inclusion criteria for the study group were: (1) sex—male gender; (2) age—18–60 years; (3) engaging directly in the woodworking process, exposed to either hardwood or softwood; (4) workers who have worked in the sawmill at least 6 months; (5) no history of smoking habit; (6) having no major chronic complications, such as tuberculosis, diabetes mellitus, hypertension, and other major respiratory illness that might interfere with the study. A group of 100 volunteers (university workers), maintaining the above criteria along with the same socioeconomic condition as the study group, was taken as a control group. Additionally, they had no history of occupational exposure to wood dust and were free from pre‐existing medical conditions, such as asthma and chronic respiratory diseases.

This study was reviewed and approved by the ethical review committee of the Department of Biochemistry and Molecular Biology (BMB) of Mawlana Bhashani Science and Technology University [Ethical approval number: MBSTU/BMB/TEST/2014/06 (2)]. All participants signed the written informed consent for participation and the use of data in the research. Moreover, the participants were given the freedom to participate in this study. There was no personally identifying information in the survey questions. All of the data were kept private and solely utilized for the purposes of this study. Before starting data collection, letters were posted to sawmill managers for getting consensus from respective sawmills.

### 2.2 Questionnaires and dust measurement

Questions were constructed by following the American Thoracic Society (ATS) and NHLI Division of Lung Diseases (DLD) questionnaire (ATS‐DLD‐78 questionnaire) standard.^23^ It included questions on demographic characteristics, respiratory complications, family history, mask using habits, and exposure years. The questionnaire was developed by visiting individual sawmills where workers were informed of the objective and given the freedom to participate in this study. Workers were asked questions, face to face, by our research members and answers were written on paper. The concentration of respirable (<5 µm), inhalable dust particles (>5 µm), and total volatile organic compounds (TVOCs) were determined by a portable air quality detector (D9 Digital Air Quality Detector; Shenzhen TOMTOP Technology Co., Ltd.).

### 2.3 Oximetry and spirometry

The individual was asked to perform both the oximetry and spirometry test after they had finished the questionnaire part. In the oximetry test, the individual was requested to put one of the fingers into an oximeter (AiQURA AD805 Finger plus oximeter) to get both heart rate and oxygen saturation level in the blood.

A portable spirometer (SP 10 spirometer; Contec Medical System Co., Ltd.) for measuring lung capacity was taken to sawmill workers and controls. Before performing spirometry, each individual was informed about the details of the spirometry process. In this study, the test was performed in a standing position, as spirometry can be performed in both sitting and standing positions, by using disposable mouthpieces and a nose clip. Test performance was repeated three times to obtain accurate lung function capacity and results were saved in the spirometer. The measurement parameters of the spirometer were: forced vital capacity (FVC), forced expiratory volume in 1 s (FEV1), the ratio of FEV1/FVC (FEV1%), peak expiratory flow (PEF), 25% flow of the FVC (FEF25), 75% flow of the FVC (FEF75), and average flow between 25% and 75% of the FVC (FEF2575).

### 2.4 Blood collection and serum separation

After performing oximetry and spirometry, 5 ml of blood were drawn from individual study subjects for further ESR, CRP, and IgE analysis. Collected blood was divided into two, 1.6 ml was kept in ESR test tubes for measuring ESR level, and the rest of the blood was kept in a serum test tube for doing both CRP and IgE tests. In the laboratory, both test tubes were kept outside the icebox to reach normal environment temperature. Serum was separated by using a tabletop centrifuge (DSC‐200T; DIGISyatem) at 1000 rotations per minute (rpm). Separated serum after proper labeling was stored in a freezer (Thermo Scientific 907 Forma −86 Ult Freezer) at −80°C.

### 2.5 ESR test

Westergren's method was adopted for measuring blood ESR levels in this study. In this method, blood was drawn into the Westergren tube from the ESR test tube and subsequently put into Westergren stand for 60 min at room temperature. After 1 h, ESR values were measured, having seen the amount of clotted erythrocyte in mm/h.

### 2.6 CRP test

CRP test was done by latex agglutination method in which CRP Latex kit (Lab21 Health Care Ltd., Biotec) was used. Latex suspension was mixed with serum on a slide and transferred to a rotation machine at 100 rpm for 1 min. The presence or absence of visible agglutination indicates the presence or absence of CRP in the specimen. Presenting the agglutination, serum was diluted 2, 4, 8, and 16 times and the amount of CRP level was calculated, observing agglutination, by 6× number of dilution time equation. Total CRP levels were represented as mg/L.

### 2.7 IgE test

Total IgE level was measured from serum by using Human IgE ELISA Kit (AMEDA Labordiagnostik GmbH). The procedure for detecting IgE levels was followed as per the user manual of the supplier. Wells were washed properly by a Microplate Washer (Thermo Scientific Wellwash) and absorbance was measured by a Microplate reader (Thermo Scientific Multiskan FC).

### 2.8 Statistical analysis

Statistical analysis was done by Statistical Packages for Social Sciences (SPSS for Windows, version 20; IBM Corp) software. *χ* ^2^ test was performed on qualitative variables that were derived from respiratory symptoms and the general characteristic of study groups. Additionally, an independent‐sample *t* test was performed to observe the mean difference of quantitative variables, including age, body mass index (BMI), spirometry parameters, oximetry parameters, and blood tests. These tests were two‐tailed and *p* < 0.05 was considered statistically significant.

## 3 RESULTS

### 3.1 General characteristics and respiratory symptoms in the study groups

Table 1 describes the general characteristics of the study groups. There were no significant differences in mean ages, BMI, alcohol consumption, and family history of respiratory problems between sawmill workers and controls. However, a significantly (*p* < 0.05) high proportion of sawmill workers (87%) did not use masks during work. Most of the workers (71%) had no institutional education, the rest 15%, 10%, and 4% had primary, high school, and college education, respectively. Both inhalable and respirable dust concentrations were reported to be 1.61 and 1.27 mg/m^3^, respectively, which are significantly higher in the study site than in the control site. However, the TVOC concentration did not show any differences.

**Table 1:** Demographic variables and level of exposure to dust particles.

Variables	Sawmill (*n* = 100)	Control (*n* = 100)	*p *Value
Age(years)	33.73 ± 7.71	31.74 ± 8.40	0.082^a^
BMI	21.24 ± 2.10	21.89 ± 2.33	0.092^a^
Education			<0.001^b^
Illiterate (%)	71	28	
Primary school (%)	15	12	
High school (%)	10	21	
Collage (%)	4	17	
University (%)	0	22	
Alcohol consumption (%)	15	9	0.192^b^
Family history of respiratory problems (%)	12	17	0.274^b^
Mask using (%)	13	23	0.066^b^
Inhalable dust particles (mg/m^3^)	1.61 ± 0.18	0.88 ± 0.10	<0.001^a^
Respirable dust particles (mg/m^3^)	1.27 ± 0.10	0.70 ± 0.11	<0.001^a^
TVOC (mg/m^3^)	0.42 ± 0.06	0.41 ± 0.07	0.428^a^

*Note*: Quantitative values are presented as mean ± standard deviation and qualitative values are presented as percentage.

Abbreviations: BMI, body mass index; TVOC, total volatile organic compounds.

^a^ *t* test.

^b^ *χ* ^2^ test.

The prevalence of respiratory symptoms was found significantly (*p* < 0.05) higher in sawmill workers compared to the control group. A higher proportion of sawmill workers complained of having breathlessness (32%), coughing (39%), sneezing (43%), chest tightness (30%), and itching (40%). Importantly, it can be noted that due to continuous exposure to wood particles at their workplaces, sawmill workers were highly suffering from itching (40%) and sneezing (43%). The prevalence of respiratory symptoms is illustrated in Table 2.

**Table 2:** Prevalence of respiratory symptoms in the exposed and nonexposed groups.

Variables	Sawmill (*n* = 100)	Control (*n* = 100)	*p *Value
Breathlessness (%)	32	14	0.002
Coughing (%)	39	15	<0.001
Sneezing (%)	43	23	0.003
Chest tightness (%)	30	12	0.002
Itching (%)	40	19	0.001

*Note*: *χ* ^2^ test was performed on respiratory symptoms. Values are represented as percentages.

### 3.2 Spirometry and oximetry

The average mean of spirometer test values was significantly (*p* < 0.05) lower in sawmill workers. Sawmill workers presented lower level of FVC (2.32 ± 0.47), FVC (%) (65.34 ± 10.23), FEV1 (2.35 ± 0.69), FEV1 (%) (72.21 ± 13.75), PEF (6.54 ± 2.05), PEF (%) (82.80 ± 20.19), FEV1/FVC (92.67 ± 4.62), FEF25 (5.73 ± 2.03), FEF75 (2.05 ± 0.61), and FEF2575 (3.52 ± 1.05) in contrast to the control group of FVC (3.10 ± 0.44), FVC (%) (76.95 ± 12.21), FEV1 (3.03 ± 0.35), FEV1 (%) (85.76 ± 9.88), PEF (8.62 ± 1.37), PEF (%) (104.18 ± 17.03), FEV1/FVC (96.36 ± 3.45), FEF25 (7.21 ± 1.16), FEF75 (2.80 ± 0.57), and FEF2575 (4.54 ± 0.94). Moreover, the average mean heart rate was significantly (*p* < 0.05) higher in sawmill workers (84.88 ± 14.91) compared with the control group (76.47 ± 9.72). However, an insignificant difference in oxygen saturation (SpO_2_) between sawmill workers and the control group was found. Table 3 elucidates both spirometer and oximeter test results.

**Table 3:** Spirometry and oximetry values between sawmill workers and control.

Variables	Sawmill workers (mean ± SD)	Control (mean ± SD)	*p *Value
FVC (L)	2.32 ± 0.47	3.10 ± 0.44	<0.001
FVC (%)	65.34 ± 10.23	76.95 ± 12.21	<0.001
FEV1 (L)	2.35 ± 0.69	3.03 ± 0.35	<0.001
FEV1 (%)	72.21 ± 13.75	85.76 ± 9.88	<0.001
PEF (L/s)	6.54 ± 2.05	8.62 ± 1.37	<0.001
PEF (%)	82.80 ± 20.19	104.18 ± 17.03	<0.001
FEV1/FVC (%)	92.67 ± 4.62	96.36 ± 3.45	<0.001
FEF25 (L/s)	5.73 ± 2.03	7.21 ± 1.16	<0.001
FEF75 (L/s)	2.05 ± 0.61	2.80 ± 0.57	<0.001
FEF2575 (L/s)	3.52 ± 1.05	4.54 ± 0.94	<0.001
Heart rate (bpm)	84.88 ± 14.91	76.47 ± 9.72	<0.001
Oxygen saturation (%)	97.94 ± 2.37	98.34 ± 1.02	0.124

*Note*: *t* Test was performed on spirometry and oximetry parameters. Values are represented as mean ± standard deviation.

Abbreviations: FVC, forced vital capacity; FEV1, forced expiratory volume in 1 s; FEV1%, the ratio of FEV1/FVC; PEF, peak expiratory flow; FEF25, 25% flow of the FVC; FEF75, 75% flow of the FVC; FEF2575, average flow between 25% and 75% of the FVC.

### 3.3 Blood test

In our study, sawmill workers had a significantly (*p* < 0.05) higher level of blood IgE (290.90 ± 39.49) compared with the control group (120.95 ± 23.00). In contrast, a slightly augmented level of ESR was also observed in sawmill workers (11.85 ± 5.40), but that level was not statistically significant in the control group (10.65 ± 4.52). In the case of CRP, however, all participants in both the sawmill workers and the control group had a blood value less than 6mg/L (Table 4).

**Table 4:** The results of blood parameters.

Variables	Sawmill workers	Control	*p *Value
IgE (IU/ml)	290.90 ± 39.49	120.95 ± 23.00	<0.001
ESR (mm/h)	11.85 ± 5.40	10.65 ± 4.52	0.090
CRP (mg/L)	All <6	All <6	

*Note*: *t* Test was performed on spirometry and oximetry parameters. Values are represented as mean ± standard deviation.

Abbreviations: CRP, C‐reactive protein; ESR, erythrocyte sedimentation rate; IgE, immunoglobulin E.

### 3.4 Spirometer parameters and exposure years

**Figure 1:**
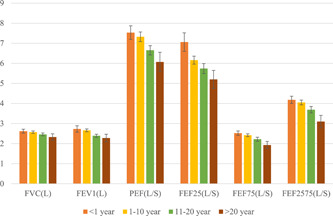
Mean of spirometer parameters according to exposure years. FVC, forced vital capacity; FEV1, forced expiratory volume in 1 s; FEV1%, the ratio of FEV1/FVC; FEF25, 25% flow of the FVC; FEF75, 75% flow of the FVC; FEF2575, average flow between 25% and 75% of the FVC; PEF, peak expiratory flow.

Figure 1 indicates a decrease in the mean of spirometer parameters, including FVC, FEV1, PEF, FEF25, FEF75, and FEF2575, with increasing exposure years of wood dust particles. From our study, it was revealed that spirometer values were higher in <1 exposure year, but low in >20 exposure years.

## 4 DISCUSSIONS

Occupational exposure to wood dust can involve significant impairment of lung functions since it is a more vulnerable internal organ. Despite knowing this hard reality, workers continuously expose themselves to these tiny particles; safety precautions, such as using masks, installing dust suck machines, and having a proper ventilation system, are not followed, which worsens respiratory health conditions. In this study, there was no significant difference in demographic variables between the two groups except education level. The average concentration of both inhalable and respirable dust was higher in study sites than in control sites. Similar findings have been found in other studies.^7^, ^15^, ^24^, ^25^ However, the concentration of dust in our study is lower than the occupational safety and health administration recommended 5 mg/m^3^ for 8 h. The most plausible explanation is that the study sites, being open sheds, have natural ventilation, which helps disperse the dust quickly. Moreover, gravimetric machines are widely used to measure both soft and hardwood dust concentrations, but we used a portable air quality detector that could measure PM2.5 and PM10 dust particles.

This study found significant and prevalent respiratory symptoms, such as breathlessness (32%), coughing (39%), sneezing (43%), chest tightness (30%), and itching (40%), in sawmill workers than in controls. The results of this present study are consistent with the previous studies.^7^, ^26^, ^27^, ^28^, ^29^ A study subjects conducted by Bislimovska et al.^8^ showed that the prevalence of cough (29.7%) and phlegm (16.2%) in the wood dust exposed group was significantly higher than in the control group. Furthermore, Hosseini et al.^30^ found a significantly higher prevalence of respiratory symptoms, including cough (40.2%), phlegm (40.6%), chest tightness (38%), and wheezing (25.3%) in the exposed group compared to the reference group. In contrast, according to Borm et al.,^31^ workers exposed to wood dust concentrations of more than 5 mg/m^3^ did not show significant respiratory symptoms. Moreover, a study on joss stick workers suggested that exposure to wood dust is not responsible for showing respiratory symptoms.^32^ This discrepancy could happen due to differences in dust concentration, work experience, types of fungal and bacterial contaminants, humidity, statistical analysis, quality of wood dust (hard, soft, allergenic, or nonallergenic), and confounding variables (controlled or not controlled).^25^

Spirometry is a widely used process to evaluate respiratory health. Importantly, in our study, spirometry parameters, such as FVC, FEV1, PEF, FEV1/FVC, FEF25, FEF75, and FEF2575, were significantly (*p* < 0.05) decreased in sawmill workers compared with controls. One study conducted on sawmills in India revealed that the spirometer parameters, including FVC, FEV1, FEF25%, slow vital capacity, peak expiratory flow rate, FEF50%, and maximum voluntary ventilation, were significantly lower in sawmill workers than in the reference group.^18^ Moreover, decreased values of FVC, FEV1, FEV1/FVC, MEF25, MEF50, MEF75, and MEF2575 among wood dust exposed workers have been documented by Bislimovska et al.^8^ and Boskabady et al.^33^ Furthermore, other studies also revealed low level of spirometer values during the study on sawmills workers.^7^, ^19^, ^34^, ^35^, ^36^ Some research works, on the other hand, are not in agreement with the findings of this present study. These studies found that the lung function of exposed workers was normal.^31^, ^37^, ^38^ For example, Bohadana et al.^37^ performed a study on oak dust‐exposed workers and found that lung function parameters were not decreased. In addition, the exposed workers in a study conducted by Borm et al.^31^ showed normal FVC and FEV1 readings. The worsening of lung health can be exacerbated by increased exposure times. According to our study high level of FVC, FEV1, PEF, FEV1/FVC, FEF25, FEF75, and FEF2575 was obtained for a <1‐year exposed group, but less in a >20‐year exposed group. Similarly, a study operated by Omole et al.^34^ found that FVC and FEV1 were significantly higher in the 1–5‐year group and low in the above 10‐year group. Hosseini et al.^30^ reported a significant difference between FVC and FEV1/FVC between duration of employment <15 years and the duration of employment ≥15 years. In contrast, in the study of Bohadana et al., as exposure time increased, the lung function parameters did not decline. Different results on pulmonary function parameters in various studies might be due to dust concentration, working environment, types of wood dust, physiological conditions, age, and working experience.

Total serum IgE level may associate with developing bronchial asthma. In sawmill workers, serum IgE levels can be augmented due to regular contact with allergenic substances, such as wood dust, endotoxin, fungi, and Gram‐negative bacteria.^39^ Significantly, the total IgE level in sawmill workers was higher in this study. This finding assents with the study by Campo et al.^40^ where it was found that wood dust sensitization was associated with a high level of total serum IgE. Additionally, an investigation of pine woodworkers in Denmark showed that only a few workers produced specific IgE against pine wood.^41^ Since higher IgE levels may have been associated with other causes, further investigations are needed for finding specific IgE for sawmill hazards. On the other hand, both ESR and CRP levels did not show any significant difference between the exposed group and the reference group. This may be due to not having antigenic properties of wood dust‐like viruses and bacteria. In contrast, a study conducted by Bhat and D'Souza^42^ documented that sawmill workers showed an augmented level of CRP than non‐sawmill workers. However, until now, there is no sufficient evidence about the association of blood ESR and CRP levels with wood dust exposure. Therefore, more studies are needed in this aspect.^43^

## 5 LIMITATIONS

Apart from the significance of our current study, it has also some limitations. First, this study has a small sample size (100 individuals in each group), making it difficult to reach conclusions. A larger study with more samples from different locations in Bangladesh could better represent the situation of the sawmill workers. Second, workers living in different zones in Bangladesh may have different socioeconomic conditions, health risks, and behaviors that might interfere with this present study. Third, the assessment of respiratory symptoms was made based on self‐report and was not validated using medical data.

## 6 CONCLUSIONS

The present study ensures and suggests that sawmill workers in Bangladesh exposed to wood dust increase the risk of developing respiratory symptoms including breathlessness, coughing, sneezing, chest tightness, and itching. Moreover, exposure to wood dust can decrease the pulmonary function of sawmill workers. A high level of total serum IgE might have been associated with hypersensitivity reaction to sawmill hazards that might be used as an accessory tool along with a spirometry test for early detection of lung function abnormalities in sawmill workers.

## AUTHOR CONTRIBUTIONS

**Md. Roman Mogal**: Conceptualization; formal analysis; investigation; methodology; writing—original draft. **Md. Didarul Islam**: Formal analysis; investigation; methodology. **Md. Ikbal Hasan**: Formal analysis; investigation; methodology. **Asadullah Junayed**: Investigation. **Sagarika Adhikary Sompa**: Investigation. **Md. Rashel Mahmod**: Investigation. **Aklima Akter**: Investigation. **Md. Zainul Abedin**: Writing—review and editing. **Md. Asaduzzaman Sikder**: Conceptualization; supervision; writing—review and editing.

## TRANSPARENCY STATEMENT

The corresponding author (Md. Asaduzzaman Sikder) confirms that this manuscript is an honest, accurate, and transparent account of the study being reported that no important aspects of the study have been omitted and that any discrepancies from the study as planned have been explained.

## Data Availability

The data sets used and analyzed during the current study are available on reasonable request from the corresponding author.
